# Duplex Interpenetrating-Phase FeNiZn and FeNi_3_ Heterostructure with Low-Gibbs Free Energy Interface Coupling for Highly Efficient Overall Water Splitting

**DOI:** 10.1007/s40820-023-01066-w

**Published:** 2023-04-10

**Authors:** Qiuxia Zhou, Caixia Xu, Jiagang Hou, Wenqing Ma, Tianzhen Jian, Shishen Yan, Hong Liu

**Affiliations:** 1 Institute for Advanced Interdisciplinary Research (iAIR), Spintronics Institute, Collaborative Innovation Center of Technology and Equipment for Biological Diagnosis and Therapy in Universities of Shandong, School of Chemistry and Chemical Engineering, University of Jinan, Jinan, 250022 People’s Republic of China; 2 School of Medical Information and Engineering, Southwest Medical University, Luzhou, 646000 People’s Republic of China; 3 Kyiv College at Qilu University of Technology, Qilu University of Technology, Shandong Academy of Sciences, Jinan, 250353 People’s Republic of China; 4 State Key Laboratory of Crystal Materials, Shandong University, Jinan, 250100 People’s Republic of China

**Keywords:** Heterostructure, Interface effect, Dealloying, Bifunctional electrocatalyst, Overall water splitting

## Abstract

The sluggish kinetics of both hydrogen evolution reaction (HER) and oxygen evolution reaction (OER) generate the large overpotential in water electrolysis and thus high-cost hydrogen production. Here, multidimensional nanoporous interpenetrating-phase FeNiZn alloy and FeNi_3_ intermetallic heterostructure is in situ constructed on NiFe foam (FeNiZn/FeNi_3_@NiFe) by dealloying protocol. Coupling with the eminent synergism among specific constituents and the highly efficient mass transport from integrated porous backbone, FeNiZn/FeNi_3_@NiFe depicts exceptional bifunctional activities for water splitting with extremely low overpotentials toward OER and HER (*η*_1000_ = 367/245 mV) as well as the robust durability during the 400 h testing in alkaline solution. The as-built water electrolyzer with FeNiZn/FeNi_3_@NiFe as both anode and cathode exhibits record-high performances for sustainable hydrogen output in terms of much lower cell voltage of 1.759 and 1.919 V to deliver the current density of 500 and 1000 mA cm^−2^ as well long working lives. Density functional theory calculations disclose that the interface interaction between FeNiZn alloy and FeNi_3_ intermetallic generates the modulated electron structure state and optimized intermediate chemisorption, thus diminishing the energy barriers for hydrogen production in water splitting. With the merits of fine performances, scalable fabrication, and low cost, FeNiZn/FeNi_3_@NiFe holds prospective application potential as the bifunctional electrocatalyst for water splitting.

**Supplementary Information:**

The online version contains supplementary material available at 10.1007/s40820-023-01066-w.

## Highlights

- Free-standing bimodal porous interpenetrating-phase FeNiZn and FeNi_3_ intermetallic heterostructure is in situ built on NiFe foam by dealloying way.
- FeNiZn/FeNi_3_@NiFe exhibits superior electrocatalytic activities toward both oxygen evolution reaction (OER) and hydrogen evolution reaction (HER) with very low overpotentials and prolonged catalytic durability in alkaline medium.
- The strong synergy between FeNiZn alloy and FeNi_3_ intermetallic generates modulated electron structure and thus well optimizes the intermediates chemisorption toward OER and HER.

**Supplementary Information:**

The online version contains supplementary material available at 10.1007/s40820-023-01066-w.

## Introduction

Electrochemical water splitting in alkaline solution represents the most promising green technique to the production of clean and sustainable hydrogen energy from the intermittent electricity harvested from renewable sources [1]. However, the kinetic limitations of the water electrolysis due to the sluggish hydrogen evolution reaction (HER) and oxygen evolution reaction (OER) result in the extra reaction overpotential, which cause excessive power consumption and high hydrogen production cost [2–4]. In view of the practical application of water splitting technique, the development of bifunctional electrocatalysts for both HER and OER can simplify the operation procedure and reduce the processing cost on account of the same fabrication technology. In this regard, ever-increasing endeavors have been made to exploring highly efficient and low-cost bifunctional electrocatalysts with the aim to facilitate the broad application of water electrocatalysis to hydrogen production.

Among diverse categories of electrocatalysts toward water splitting, intermetallics as one particular class of alloy-type materials have attracted widespread attentions in recent years [5, 6]. Intermetallics possess the chemical interactions of ionic or covalent combination between the well-defined stoichiometric compositions, which can effectively restrain the surface atom migration and reconstruction compared with the monometal and substitutional alloys [5]. Owing to the specific bonding features, intermetallic compounds often take on superior catalytic activity and resistance for oxidation and dissolution in electrolyte [7]. Noticeably, a variety of Ni-based nonprecious intermetallics have been discovered to own outstanding intrinsic catalytic activity toward water splitting, such as NiAl [8], MoNi_4_ [9], FeNi_3_ [10, 11], CuNNi_3_ [12] toward OER, and Ni-Mo [13, 14], Ni–Ti [15, 16], and NiZn [17, 18] toward HER. Even so, for the single intermetallic itself, almost no one has the bifunctional activities toward water splitting. Combining intermetallic with other materials represents one of the effective tactics to create unique OER activity as well as superior HER activity. Recently, some FeNi_3_-based nanocomposites have been focused to achieve the exceptional bifunctional catalytic activities. For instance, composited Ni_3_FeN/Ni_3_Fe nanosheets exhibits high bifunctional activities with the overpotentials of 250 and 125 mV for OER and HER to drive a current density of 10 mA cm^−2^ [19]. Liang et al. fabricated the lamellar FeNi_3_N incorporated by FeNi_3_, which displayed the *η*_10_ of 260 and 51 mV toward OER and HER, respectively [20]. Compared with the single counterpart, the formation of the FeNi_3_-based nanocomposites can significantly improve the bifunctional catalytic activities because the electronic interaction between the components can optimize the electronic structure, balance the adsorption and desorption of the reaction intermediates, as well increase the number of the active sites [19, 20]. Motivated by these reports, it is appealing to further screen high-performance bifunctional FeNi_3_-based nanocomposite toward water splitting by way of tailoring the constituents and architecture.

Of note, the architecture of catalyst electrode is also one of pivotal factor to decrease the overpotential of water electrolysis not only by providing the abundant active sites and the fluent diffusion path but also eliminating a series of inherent resistance among the catalyst surface, electrolyte, and the electrode itself. In this respect, self-supporting electrode is preferable for exposing more active sites and reducing the interface resistance owing to free of conductive binders [21–23]. Recently, the in situ construction of nanostructured electrocatalysts on bulky metal foam has been one powerful strategy to improve the efficacy of overall water splitting [24–26]. For example, Wang et al. prepared the IrNi-FeNi_3_ hybrid supported on nickel foam (IrNi-FeNi_3_/NF), which displays the ultralow overpotentials of 330.0/288.8 mV to generate a current density 1000 mA cm^−2^ for OER and HER as well good stability for overall water splitting [26]. Such the unique nanosheets structure anchored on three-dimensional (3D) nickel foam exposed more catalytic active sites and facilitated the mass transfer. It should be mentioned that among various nanostructures, 3D porous structure has been witnessed to possess inherent structural superiorities in the electrocatalysis in terms of the robust structure stability and the maximized exposure of surface-active sites [27, 28]. Furthermore, the in situ construction of nanoporous electrocatalysts over macroporous metal foam can generate highly efficient hierarchical porous architecture toward electrocatalysis, where the large pores from support can provide unblocked electrolyte penetration along the interconnected framework and hollow channels; meanwhile, the nanoscale porous surface can furnish high density of active sites and full contact with the electrolyte [29–31]. In addition, the integrated interconnected multidimensional skeleton can prevent the structure from aggregation as well as retard the corrosion and dissolution of catalyst materials in electrolyte under high applied potential.

Given all that, in current work we focused on the design and fabrication of high-performance bifunctional interpenetrating-phase FeNiZn and FeNi_3_ intermetallic heterostructure in situ constructed within the surface layer of NiFe foam (FeNiZn/FeNi_3_@NiFe) with multilevel pore distribution. NiFe foam of the atomic ratio around 80:20 was chosen as the support with the phase structure consisting of FeNi_3_ intermetallic and pure Ni. The versatile dealloying strategy was deployed to simultaneously in situ build one layer of bimodal nanoporous structure and dispersed FeNiZn alloy within NiFe foam through the handy operation of electroplating Zn, annealing, and etching part of Zn. The as-made FeNiZn/FeNi_3_@NiFe gets together multiple structural advantages in terms of multimodal porous structure from macro- to nanoscale and the rich interface between interpenetrating FeNiZn and FeNi_3_, thus resulting in unlimited mass transport and high density of active sites. First-principle calculations testified that FeNiZn and FeNi_3_ heterostructure has diversified highly active sites with low-Gibbs free energy toward OER and HER. The as-built water electrolyzer achieves record-high stable performances for hydrogen production under much lower cell voltage even compared with the previously reported similar noble metal-containing catalysts, such as IrNi-FeNi_3_/NF [26]. This work can provide new avenues for screening the self-supporting intermetallic-based bifunctional electrocatalysts with high performances, easy preparation, and economic cost.

## Experimental Sections

### Chemicals

Commercial Pt/C (20 wt%) and RuO_2_ were obtained from Sigma Aldrich Chemical Reagent Co., Ltd. The other chemicals were bought from Shanghai Sinopharm Chemical Reagent Ltd. Co of China. All agents were used as an analytical purity without further purification.

### Preparation of FeNiZn/FeNi_3_@NiFe

NiFe foam with the size around 1 cm × 2 cm was pretreated by ultrasonicating in 1 M HCl solution, ethanol, and deionized water, respectively. Zn coating was electrodeposited on NiFe foam to form Zn@NiFe in the mixture solution of 0.2 M ZnSO_4_ + 0.2 M Na_2_SO_4_ + 0.2 M H_3_BO_3_ in a three-electrode system with Pt sheet (2 cm × 3 cm) and saturated calomel electrode (SCE) as the counter and reference electrode under − 1.7 V versus SCE for 1 h. Subsequently, Zn@NiFe was annealed at 430 °C for 5 h in the Ar atmosphere to make the alloy precursor, which was further corroded in 2.0 M ammonia solution for 12, 24, and 48 h to obtain the final FeNiZn/FeNi_3_@NiFe-12 h, FeNiZn/FeNi_3_@NiFe-24 h, and FeNiZn/FeNi_3_@NiFe-48 h samples, respectively.

### Characterization

The crystal phase structure of the sample was determined by X-ray diffraction (XRD, Bruker D8 advanced) with Cu Kα radiation (*λ* = 1.5418 Å) at a step rate of 0.02° s^−1^. The morphology and composition were characterized by transmission electron microscope (TEM, JEOL JEM-F200) and field-emission scanning electron microscope (SEM, Hitachi Regulus 8100) equipped with X-sight energy-dispersive X-ray spectrometer (EDS, Oxford INCA). Electron backscattered diffraction (EBSD) was conducted by a field-emission SEM (FEI Scios) using an OXFORD EBSD acquisition system. X-ray photoelectron spectroscopy (XPS) data were recorded on an ESCALAB MK II X-ray photoelectron spectrometer. The pore size distribution of the sample was measured with a Quadrasorb SI-MP (Quantachrome Instruments) according to the Brunauer-Emmett-Teller (BET) method.

### Electrochemical Measurements

All electrochemical data were recorded on CHI 760E electrochemical workstation (Shanghai Chenhua Co., China) in a three-electrode system. The electrocatalysts were employed as the working electrode with the fixed size at ~ 0.2 cm^2^ (0.4 cm × 0.5 cm). Graphite rod and mercuric oxide electrode (MOE) were selected as the counter electrode and reference electrode, respectively. The electrolyte of 1.0 M KOH (pH = 14) solution was first purged with N_2_ and O_2_ for 30 min before HER and OER testing, respectively. The linear sweep voltammetry (LSV) was conducted with the scan rate of 5 mV s^−1^. All the potentials were converted to reversible hydrogen electrode (RHE) according to the equation of *E*_RHE_ = *E*_MOE_ + 0.924 V with 90% iR-compensation. The double-layer capacitance (*C*_dl_) values were confirmed by cyclic voltammetry (CV) in the potential range of 1.00 to 1.10 V (vs. RHE) at different scan rates. Electrochemical impedance spectroscopy (EIS) was measured at the overpotential of 100 and 250 mV for HER and OER with a frequency scope from 100 kHz to 0.01 Hz, respectively. Pt/C and RuO_2_ were dropped onto NiFe foam (0.4 cm × 0.5 cm) with the loading mass of ~ 3.5 mg cm^−2^ for all tests, respectively. Current–time (*i*–*t*) curves were tested under constant potentials.

### Density Functional Theory Calculations

Spin-polarized first-principle calculations were performed by the density functional theory (DFT) using the Vienna Ab-initio Simulation Package (VASP) package [32]. The generalized gradient approximation (GGA) with the Perdew–Burke–Ernzerhof (PBE) functional was used to describe the electronic exchange and correlation effects [33–35]. Uniform G-centered *k*-points meshes with a resolution of 2*π* × 0.03 Å^−1^ and Methfessel–Paxton electronic smearing were adopted for the integration in the Brillouin zone for geometric optimization. The simulation was run with a cutoff energy of 500 eV throughout the computations. These settings ensure convergence of the total energies to within 1 meV per atom. Structure relaxation proceeded until all forces on atoms were less than 1 meV Å^−1^ and the total stress tensor was within 0.01 GPa of the target value.

The free energy of the adsorption atomic hydrogen (Δ*G*_H*_) is obtained by:

1 $$\Delta G_{{{\text{H}}^*}} = \Delta E_{{{\text{H}}^*}} + \Delta E_{{{\text{ZPE}}}} - T\Delta S_{{{\text{H}}^*}}$$

Δ*E*_H*_ describes the energy needed to increase the coverage by one hydrogen atom. Δ*E*_ZPE_ is the difference in zero point energy and Δ*S*_H*_ is the difference in entropy. Δ*E*_ZPE_–*T*Δ*S*_H*_ is about 0.24 eV, so Δ*G*_H*_ = Δ*E*_H*_ + 0.24 [36]. For Δ*E*_H*_, it is calculated as follows:

2 $$\Delta E_{{{\text{H}}^*}} = E({\text{surface}} + H) - E({\text{surface}}) - \frac{1}{2}E(H_{2} )$$

where *E*(surface + *H*) represents the total energy of the selected surfaces with one adsorbed hydrogen atom on the surfaces, *E*(surface) represents the total energy of the surfaces, while *E*(*H*_2_) represents the total energy of a gas phase *H*_2_ molecule.

Generally, in alkaline media the OER reaction mechanism can be written as 4-elecetron change mechanism:

- Step 1: $${}^* + {\text{OH}}^{ - } \to {\text{OH}}^{*} + {\text{e}}^{ - }$$
- Step 2: $${\text{OH}}^{*} + {\text{OH}}^{ - } \to {\text{O}}^{*} + {\text{H}}_{2} {\text{O}} + {\text{e}}^{ - }$$
- Step 3: $${\text{O}}^{*} + {\text{OH}}^{ - } \to {\text{OOH}}^{*} + {\text{e}}^{ - }$$
- Step 4: $${\text{OOH}}^{*} + {\text{OH}}^{ - } \to {\text{O}}_{2} ({\text{g}}) + {}^{*} + {\text{H}}_{2} {\text{O}} + {\text{e}}^{ - }$$

where * presents an adsorption site on the catalyst and OH*, O*, and OOH* denote the corresponding absorbed intermediates. Accordingly, the free energy of reactions (S1)–(S4) can be calculated using aforementioned equations:

$$\Delta G1 = G({\text{OH}}^{*} ) - G(*) - G({\text{OH}}^{ - } )$$

$$\Delta G2 = G({\text{O}}^{*} ) + G({\text{H}}_{2} {\text{O}}) - G({\text{OH}}^{*} ) - G({\text{OH}}^{ - } )$$

$$\Delta G3 = G({\text{OOH}}^{*} ) - G({\text{O}}^{*} ) - G({\text{OH}}^{ - } )$$

$$\Delta G4 = 4.92 - \Delta G1 - \Delta G2 - \Delta G3$$

The theoretical overpotential *η* for OER can be calculated using Eqs. 3 and 4:

3 $$G^{{{\text{OER}}}} = {\text{max}}\left\{ {\Delta G1,\Delta G2,\Delta G3,\Delta G\left. 4 \right\}} \right.$$

4 $$\eta^{{{\text{OER}}}} = {{G_{{{\text{OER}}}} } \mathord{\left/ {\vphantom {{G_{{{\text{OER}}}} } e}} \right. \kern-0pt} e} - {1}{\text{.23 V}}$$

## Results and Discussion

### Preparation and Characterization

**Fig. 1:**
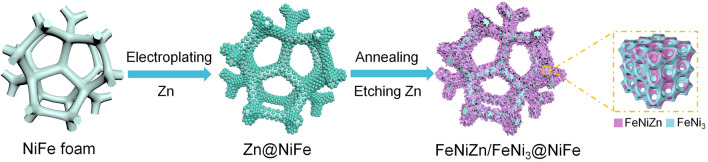
Preparation scheme of FeNiZn/FeNi_3_@NiFe

The interpenetrating FeNiZn and FeNi_3_ heterojunction on NiFe foam was fabricated as shown in Fig. 1. NiFe foam with the atomic ratio around 80:20 was chosen as the conductive support, which has the interconnected macroscale skeleton and hollow channels with the pore size around 150–200 μm as shown in Fig. S1a, b. One layer of dense zinc film is electrodeposited on the surface of the NiFe foam (Zn@NiFe) and then annealed in the inert atmosphere. Subsequently, a part of Zn atoms are selectively dissolved in ammonia solution to get the target product (FeNiZn/FeNi_3_@NiFe).

**Fig. 2:**
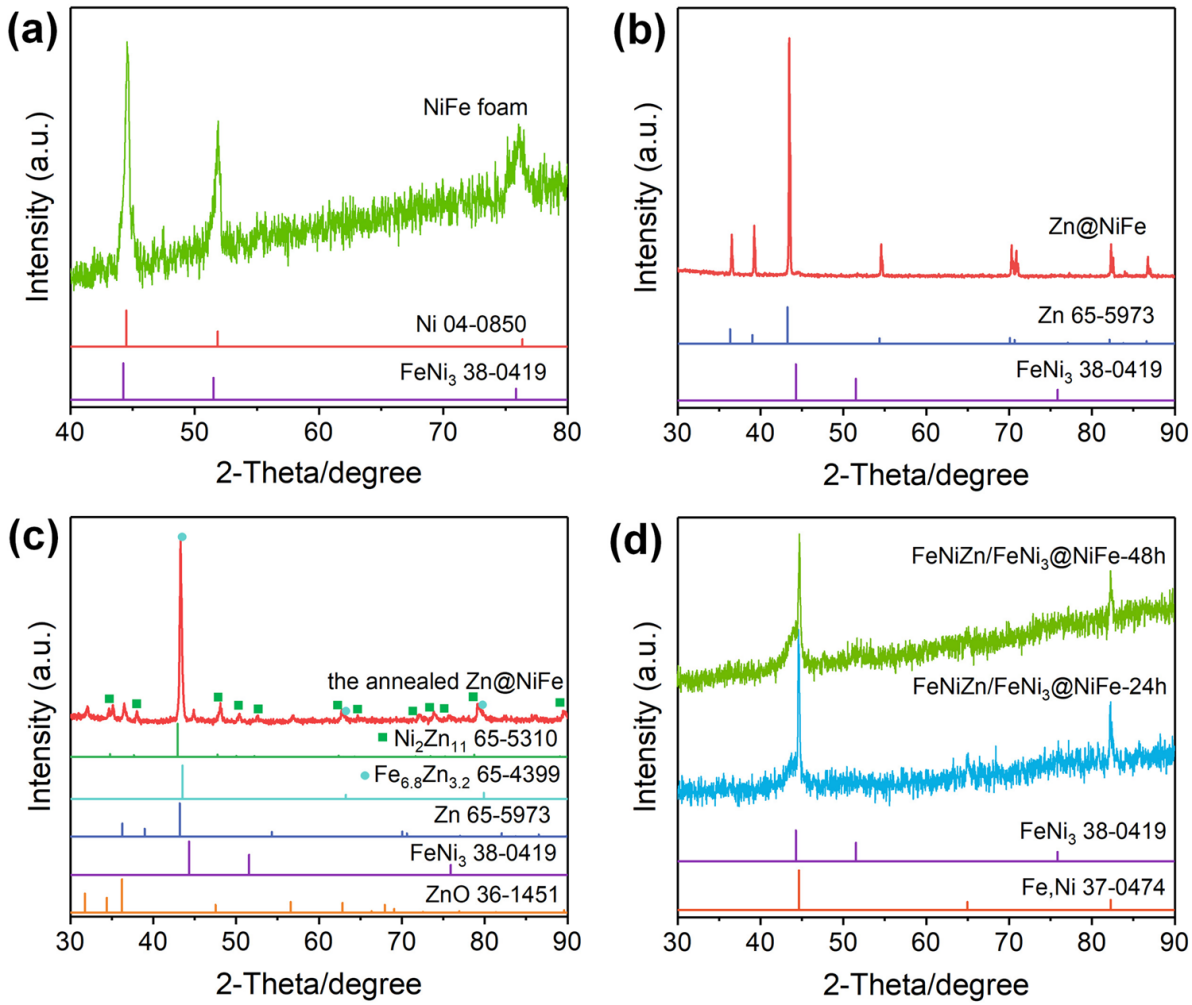
XRD patterns of **a** NiFe foam, **b** Zn@NiFe sample by electroplating Zn on NiFe foam, **c** the annealed Zn@NiFe sample, and **d** FeNiZn/FeNi_3_@NiFe samples by dealloying in 2.0 M ammonia solution for 24 and 48 h

**Fig. 3:**
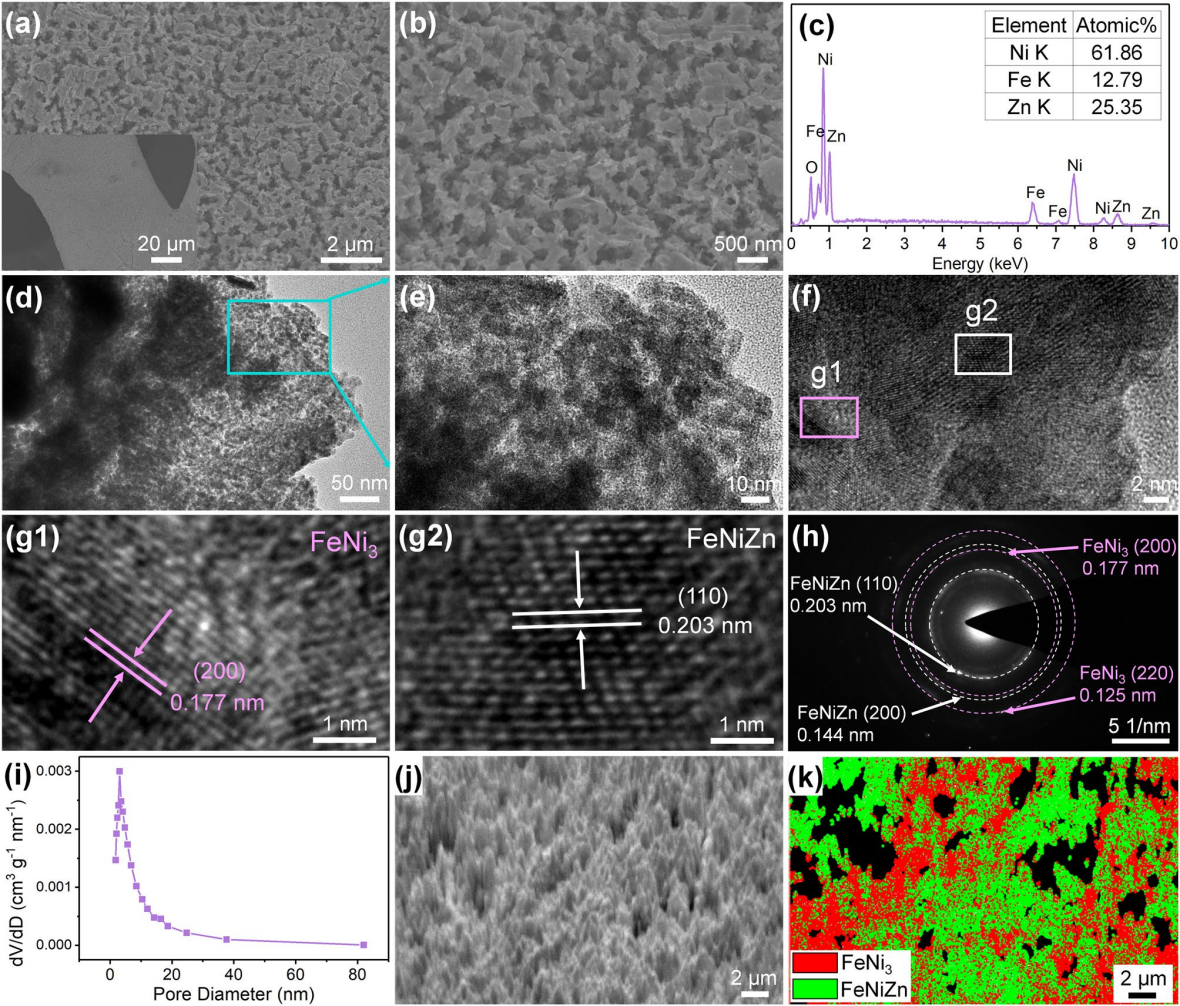
**a**, **b** SEM images, **c** EDS data, **d**, **e** TEM images, **f**, **g** HRTEM images, **h** SAED pattern, **i** BJH pore size distribution curve, and **j, k** EBSD images of the FeNiZn/FeNi_3_@NiFe sample dealloyed in the 2.0 M ammonia solution for 24 h

XRD was first applied to examine the evolution of crystal phase structure from NiFe foam to the final product during electroplating Zn, annealing, and partial etching of Zn. For the NiFe foam, three asymmetric diffraction peaks with the evident shoulder at the lower-angle side emerged at 44.5°, 51.8°, and 76.1°, which is owing to the neighboring relationship for the diffraction peaks of pure Ni and FeNi_3_ intermetallic (Fig. 2a). Upon the electrodeposition of Zn, the sharp diffraction peaks of Zn emerged and covered the original peaks of NiFe foam (Fig. 2b). After annealing at 430 °C for 5 h, the Zn atoms diffused into the foam support to form the Ni_2_Zn_11_ and Fe_6.8_Zn_3.2_ alloy as denoted in Fig. 2c, while some Zn atoms residued at the surface along with certain oxidation. The diffraction peaks for the FeNi_3_ intermetallic still retained. Through leaching out part of Zn atoms in the ammonia solution for 24 h (Fig. 2d), FeNi_3_ phase still existed with the diffraction peaks widening, suggesting that the original bulky FeNi_3_ intermetallic converted to the nanoscale structure as a result of the strategy of Zn introduction and etching. Meanwhile, there are new three diffraction peaks that appeared around 44.6°, 65.0°, and 82.3° corresponding to the phase structure similar to that of (Fe, Ni) alloy (JCPDS No. 37-0474). In the light of the high content of Zn upon dealloying as indicated in Fig. 3c, such the species should be assigned to FeNiZn alloy. It is considered that during the high-temperature annealing the introduced Zn will combine with the Ni and a part of FeNi_3_ intermetallic in NiFe foam to form Ni_2_Zn_11_ and Fe_6.8_Zn_3.2_ alloy. After selectively dissolving the Zn atoms, the residual Fe, Ni, and Zn atoms underwent interdiffusion to form the ternary FeNiZn alloy. Note that no extra peaks emerged, which manifested the formation of FeNi_3_ intermetallic and FeNiZn alloy composites. By further extending the corrosion time to 48 h, the XRD pattern has almost no change apart from the minor variation of peak width and intensity, indicating the more stable phase structure for the FeNiZn and FeNi_3_ heterojuction.

The microstructural evolution process from NiFe foam to the resultant heterostructure was monitored by electron microscope. From Fig. S1a, b, it is clear that NiFe foam has the porous structure with interconnected pore channels and smooth 3D skeleton, which can provide high supporting area as well as the expedite pathway for mass transfer. Its atomic ratio of Ni and Fe is around 81.20:18.80 (Fig. S1g). Upon electroplating Zn on NiFe foam, one dense layer of Zn was successfully coated as observed in Fig. S1c, d and EDS data (Fig. S1h). After annealing, the coating layer became more flattening with Ni, Fe, and Zn elements detectable, implying the formation of Zn-based alloy precursor (Fig. S1e, f, and i).

The microstructure of the dealloyed FeNiZn/FeNi_3_@NiFe sample upon etching in 2.0 M ammonia solution for 24 h is shown in Fig. 3a, b. There are rich pore channels formed on the surface of 3D NiFe backbone with the size around several hundred nanometers. EDS analysis elucidated that the ratio of Ni, Fe, and Zn on the surface layer was 61.86:12.79: 25.35 (Fig. 3c). TEM images gave the detailed structure of the slice peeled off from FeNiZn/FeNi_3_@NiFe-24 h sample by long-time ultrasonicating. As illustrated in Fig. 3d, e, there are abundant pores embedded in the peeled slice with the typical size as narrow as 3 nm. In addition, the BJH analysis further supports such the microscope observation. From Fig. 3i, the second-order pores size for FeNiZn/FeNi_3_@NiFe-24 h sample mainly distributes in the range of 3 ~ 6 nm, which accords with the TEM observation. As shown by the high-resolution TEM (HRTEM) image in Fig. 3f, the ordered fringe lattices from the square frame of g1 and g2 are calculated to be 0.177 and 0.203 nm, which can be assigned to the (200) plane of FeNi_3_ intermetallic and (110) plane of FeNiZn alloy. From Fig. 3h, the diffraction rings in selected area electron diffraction (SEAD) pattern correspond to the (220) and (200) planes for FeNi_3_ and the (110) and (200) planes of FeNiZn alloy, confirming the polycrystalline nature of FeNiZn and FeNi_3_ heterojunction. EBSD images in Fig. 3j, k indicate the interpenetrating distribution between FeNiZn alloy and FeNi_3_ intermetallic, where the measured phase ratio is around 0.51:0.49. According to the elemental components and phase ratio, it can be speculated that the FeNiZn alloy ratio is 8.74:57.48:33.78. In addition, the influence of the different corrosion time on the resulting structure was explored as displayed in Fig. S3, and the detailed description was provided in Supporting Information.

**Fig. 4:**
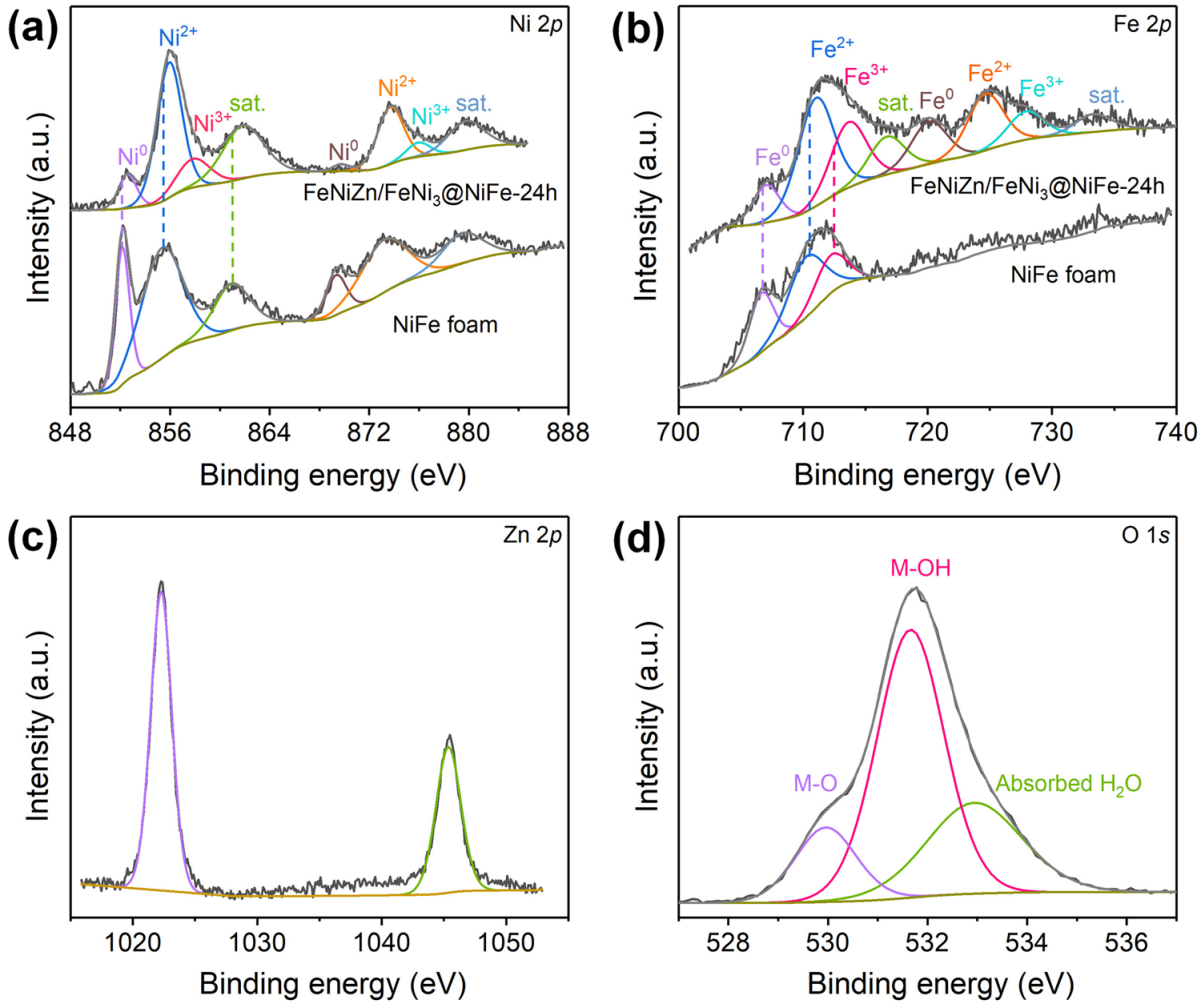
XPS data of **a** Ni 2*p*, **b** Fe 2*p*, **c** Zn 2*p*, and **d** O 1*s* in the as-made FeNiZn/FeNi_3_@NiFe-24 h sample and NiFe foam

XPS represents one forceful technique to examine the chemical combination of each element in the composite. Figure 4 gives the XPS data of the as-made FeNiZn/FeNi_3_@NiFe-24 h and pure NiFe foam. As shown in Fig. 4a, for FeNiZn/FeNi_3_@NiFe-24 h sample the peaks with the binding energy at 852.52 and 868.54 eV can be ascribed to the metallic Ni. The slight negative shift compared with the pure Ni suggests that the metallic Ni comes from the FeNiZn alloy and FeNi_3_ intermetallic [37, 38]. Moreover, the two peaks around 856.13 and 873.88 eV with the satellite peaks can be attributed to the Ni^2+^ species accompanied with the coexistence of Ni^3+^ corresponding to the fitted peaks around 858.05 and 876.15 eV [26, 39]. The analysis results above illustrate that Ni surface oxide and hydroxide formed on the sample surface during the dealloying and drying process due to the more reactive property of nanosized Ni. As for the XPS data of NiFe foam, the Ni core level shows the similar feature. By comparison, it is evident that all the binding energies for Ni species in FeNiZn/FeNi_3_@NiFe-24 h encounter the positive shift to higher value, which testifies that the addition of Zn and the interface formation effectively regulates the electronic structure state of Ni. As for the Fe 2*p* core level region (Fig. 4b), the peak at 707.03 eV stems from the metallic Fe within the FeNiZn alloy and FeNi_3_ [40]. The split peaks at 711.02, 713.64, and 716.73 eV state the existence of Fe^2+^ and Fe^3+^ resulting from the Fe oxide and hydroxide as the same as the case of Ni [19, 40]. It is noted that the binding energies for Fe species also underwent the positive shift compared with that of NiFe foam, indicating the modulated electronic state of Fe after introducing Zn and interface generation [41, 42]. For the Zn 2*p* spectrum in Fig. 4c, the peak located at 1022.29 eV belongs to the metallic Zn from FeNiZn alloy [43]. It should be mentioned that its binding energy went through relatively large positive shift by 1.12 eV in comparison to pure Zn, which is considered to originate from the electron transfer of Zn toward Fe and Ni owning to the formation of FeNiZn alloy as well as the electron interaction between FeNiZn and FeNi_3_ heterostructure. It is clear that the elements of Ni, Fe, and Zn in the heterostructure can generate distinct electron modulation. In the O 1*s* spectrum as depicted in Fig. 4d, the peaks around 529.96, 531.66, and 532.94 eV can be ascribed to the metal–O (M–O), metal–OH (M–OH), and absorbed water molecules on metals as a result of the formation of metal oxide and hydroxide, which is identical with the observations above for the formation of metal ions [44].

### Electrochemical Performances

**Fig. 5:**
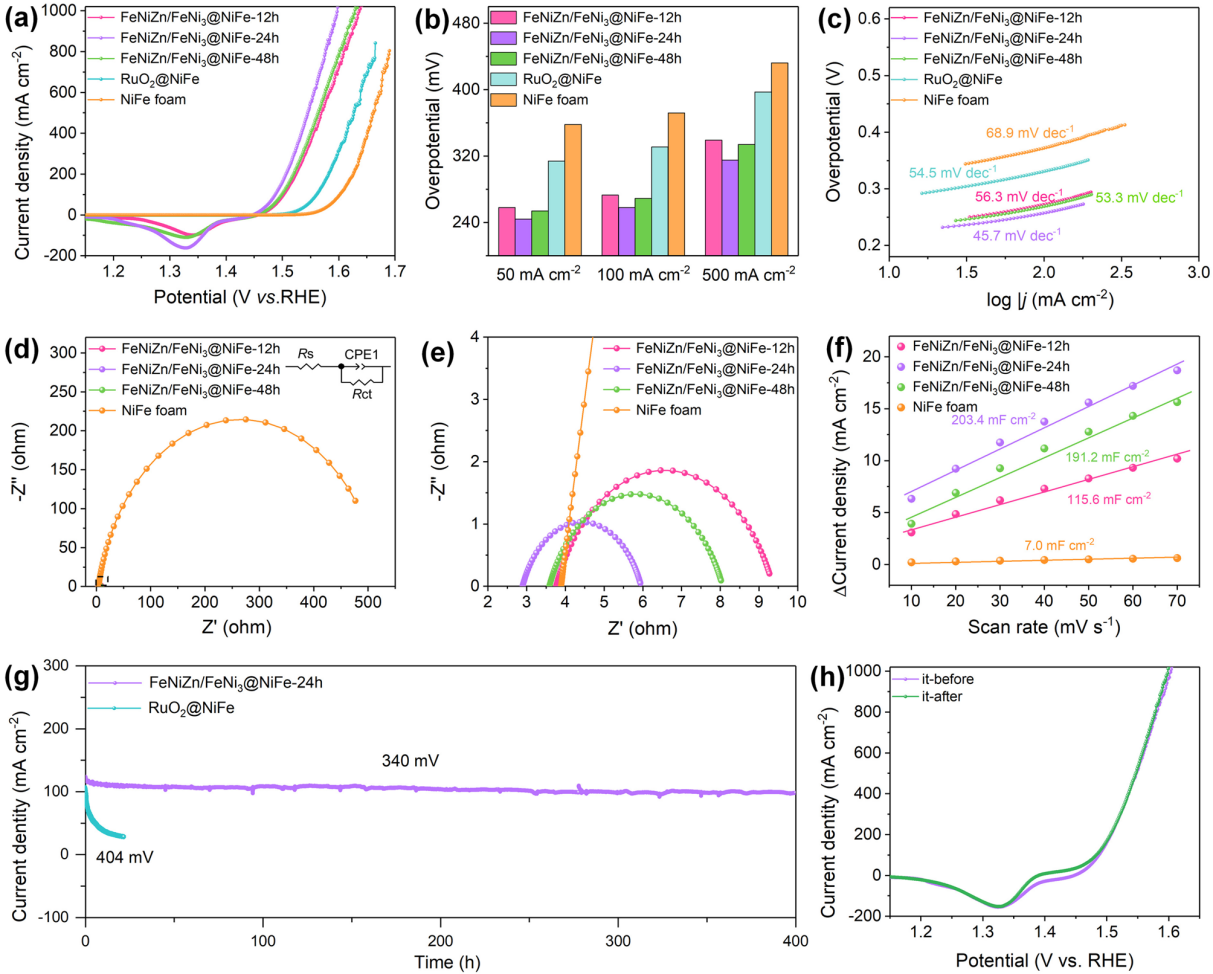
**a** LSV curves of FeNiZn/FeNi_3_@NiFe samples, RuO_2_@NiFe, and NiFe foam in 1.0 M KOH solution at the scan rate of 5 mV s^−1^. **b** Overpotentials of all electrodes at the 50, 100, and 500 mA cm^−2^. **c** Tafel plots of all samples. **d**, **e** EIS data of FeNiZn/FeNi_3_@NiFe samples and NiFe foam. **f** The capacitive current at 1.05 V vs. RHE at different scan rates. **g** *i–t* curve under the overpotential of 340 and 404 mV for FeNiZn/FeNi_3_@NiFe-24 h and RuO_2_@NiFe, respectively, and **h** LSV curves at a scan rate of 5 mV s^−1^ before and after *i*–*t* test for FeNiZn/FeNi_3_@NiFe-24 h toward OER

The OER performances of FeNiZn/FeNi_3_@NiFe samples were first accessed in 1.0 M KOH solution with those of RuO_2_/NiFe and NiFe foam included for comparison in Fig. 5a. All the LSV curves for the as-made samples with different dealloying time show one evident reduction peak in the potential range of 1.25–1.4 V, which possibly resulted from the reduction of Ni^3+^ to Ni^2+^ [45, 46]. By comparison, it is clearly found that the FeNiZn/FeNi_3_@NiFe-24 h sample takes on the supreme electrocatalytic activity toward OER with the lowest overpotentials of 367 mV to reach the high current density of 1000 mA cm^−2^. Figure 5b provides the detailed overpotentials of all electrocatalysts at different current densities. At the current density of 50 mA cm^−2^ for the FeNiZn/FeNi_3_@NiFe-24 h electrode, the overpotential is only 244 mV, which is dramatically lower than those of RuO_2_@NiFe (314 mV) and NiFe foam (358 mV). At the current density of 100 and 500 mA cm^−2^, its overpotentials are 258 and 315 mV, respectively, which is also much smaller than those of RuO_2_@NiFe (331 and 397 mV) and NiFe foam (372 and 432 mV). Such the OER activity for FeNiZn/FeNi_3_@NiFe-24 h surpasses some similar electrocatalysts reported previously as shown in Table S1 [19, 20, 24, 26, 38, 39, 47–51].

We further deduced the Tafel plots of all electrocatalysts on the basis of the LSV curves to gain insight into their OER mechanism. As depicted in Fig. 5c, the Tafel plot for FeNiZn/FeNi_3_@NiFe-24 h displays the smallest value around 45.7 mV dec^−1^ compared with other electrodes, implying that it has the relatively low reaction kinetic limitation. In an effort to understand the reaction kinetic behavior of electrode and interface, EIS analysis was conducted. As shown in Fig. 5d, e, FeNiZn/FeNi_3_@NiFe-24 h sample shows the lowest electrochemical transfer resistance (*R*_ct_) at 3.06 Ω compared with other electrodes, which means its much better electrocatalytic efficacy favoring for highly effective electron transfer and mass transport during the oxygen evolution. The *C*_dl_ value calculated from CV curves is further utilized to estimate the electrochemical surface area (ECSA) of all electrodes (Fig. S4). As can be observed in Fig. 5f, the *C*_dl_ values for all the electrodes can clearly account for their disparity in the OER activities. FeNiZn/FeNi_3_@NiFe-24 h has the *C*_dl_ value as large as 203.4 mF cm^−2^, which is much larger than that of FeNiZn/FeNi_3_@NiFe-12 h (115.6 mF cm^−2^), FeNiZn/FeNi_3_@NiFe-48 h (191.2 mF cm^−2^), and NiFe foam (7.0 mF cm^−2^). Such the large ECSA of FeNiZn/FeNi_3_@NiFe-24 h states its high density of active sites toward OER. It is regarded that the mesoporous structure with small pore size offers high surface area and thus the abundant active sites. Considering the experimental observations above, there is no doubt that the exceptional OER activity of FeNiZn/FeNi_3_@NiFe-24 h profits from the specific multidimensional porous interpenetrating-phase heterostructure between FeNiZn alloy and FeNi_3_ intermetallic. The rich mesoscale pores dispersed within the interconnected 3D skeleton can furnish high density of active sites, while the large pore channels can provide smooth electron transfer and unblocked electrolyte diffusion. In particular, the strong synergy from FeNiZn alloy and FeNi_3_ intermetallic is beneficial for the modulation of electronic structure and thus the optimization of reaction intermediates chemisorption [2, 41].

The electrocatalytic durability for one OER electrocatalyst also represents one crucial indicator for its practical application in industrial hydrogen production. Chronoamperometry method was first used to estimate the long-term persistence in water electrolysis for FeNiZn/FeNi_3_@NiFe-24 h sample. For comparison, the catalytic durability of the commercial RuO_2_ supported on NiFe foam was also provided. As illustrated in Fig. 5g, FeNiZn/FeNi_3_@NiFe-24 h electrode can steadily proceed successive oxygen evolution for 400 h under the overpotential of 340 mV without the evident activity degradation, suggesting its excellent catalytic stability toward OER. In contrast, the catalytic activity of RuO_2_@NiFe dramatically decreased with only 28% of the initial current density retained upon testing for 22 h. The obvious activity decline of RuO_2_@NiFe is mainly because such the catalyst easily dissolves in a strong alkaline electrolyte under high voltage, meanwhile the particle-type catalyst usually suffers from peeling off during long term test in comparison with the integrated self-supporting electrocatalyst [21, 52]. The outstanding electrocatalytic stability of FeNiZn/FeNi_3_@NiFe-24 h electrode toward OER was further confirmed by the well overlapped LSV curves before and after the long-term running (Fig. 5h). In view of the origin of high OER catalytic durability, the microstructural and constituent changes after electrocatalysis were in detail inspected. From the SEM image in Fig. S5a, it is clear that the porous morphology well kept, where the skeleton became more uniform and the pore channels appeared to be more thorough. More importantly, the TEM image of the sample after testing proves that the mesoporous structure within the skeleton also has no size coarsening (Fig. S5b). Furthermore, the *C*_dl_ value (214.4 mF cm^−2^) of the electrode after testing became even larger than that of pre-cycling sample, which is responsible for the stable OER activity upon long term test (Fig. S6). The slight increment of *C*_dl_ value is considered to stem from the surface reconstruction due to the further dissolution of little Zn atom as confirmed by EDS data (Fig. S5d). EDS data confirm that the ratio of Ni/Fe/Zn is 62.35:16.38:21.27 after long-term test. By comparison with that of the fresh sample, the Zn content decreased, while the contents of Fe and Ni both increased. It can be inferred that during long-term catalysis the Zn atoms encounter relatively more dissolution in KOH solution compared with Fe and Ni in the oxygen-rich environment, which also lead to the enlarged pore channels as observed in Fig. S5a, and thus generate much higher surface area as well as the increment of active sites toward OER (Fig. S6). It is considered that during the long-term measurement Fe atoms tend to combine with oxygen-containing species to form hydroxide, thus retarding its dissolution. XRD was further utilized to analyze the phase structure of the resultant sample. As shown in Fig. S5c, the FeNiZn and FeNi_3_ well maintained the initial phase, indicating its unique stability in alkaline solution under the high applied potential. The results above demonstrate that the free-standing multi-scale 3D porous heterostructure has robust structure stability, which can well preserve the surface active sites during long period of electrocatalysis.

XPS was also used to examine the chemical state of all elements for the resultant FeNiZn/FeNi_3_@NiFe-24 h sample after testing. As shown in Fig. S7a, c, the spectra of both Ni 2*p* and Zn 2*p* have almost no change compared with those of the fresh sample. In the respect of Fe 2*p* spectrum (Fig. S7b), it is evident that the peak intensity of Fe^3+^ increased along with that of Fe^2+^ diminished, which is due to the little oxidation of Fe^2+^ to Fe^3+^ during OER process. In addition, the enhanced intensity of M–OH also suggested that a little Fe^2+^ species converted to FeO–OH species (Fig. S7d). The observations above dramatically demonstrated that self-supporting multimodal porous FeNiZn and FeNi_3_ heterostructure has excellent electrocatalytic durability toward OER in virtue of the robust structure stability as well as the high corrosion resistance, demonstrating powerful application prospect in water electrocatalysis.

**Fig. 6:**
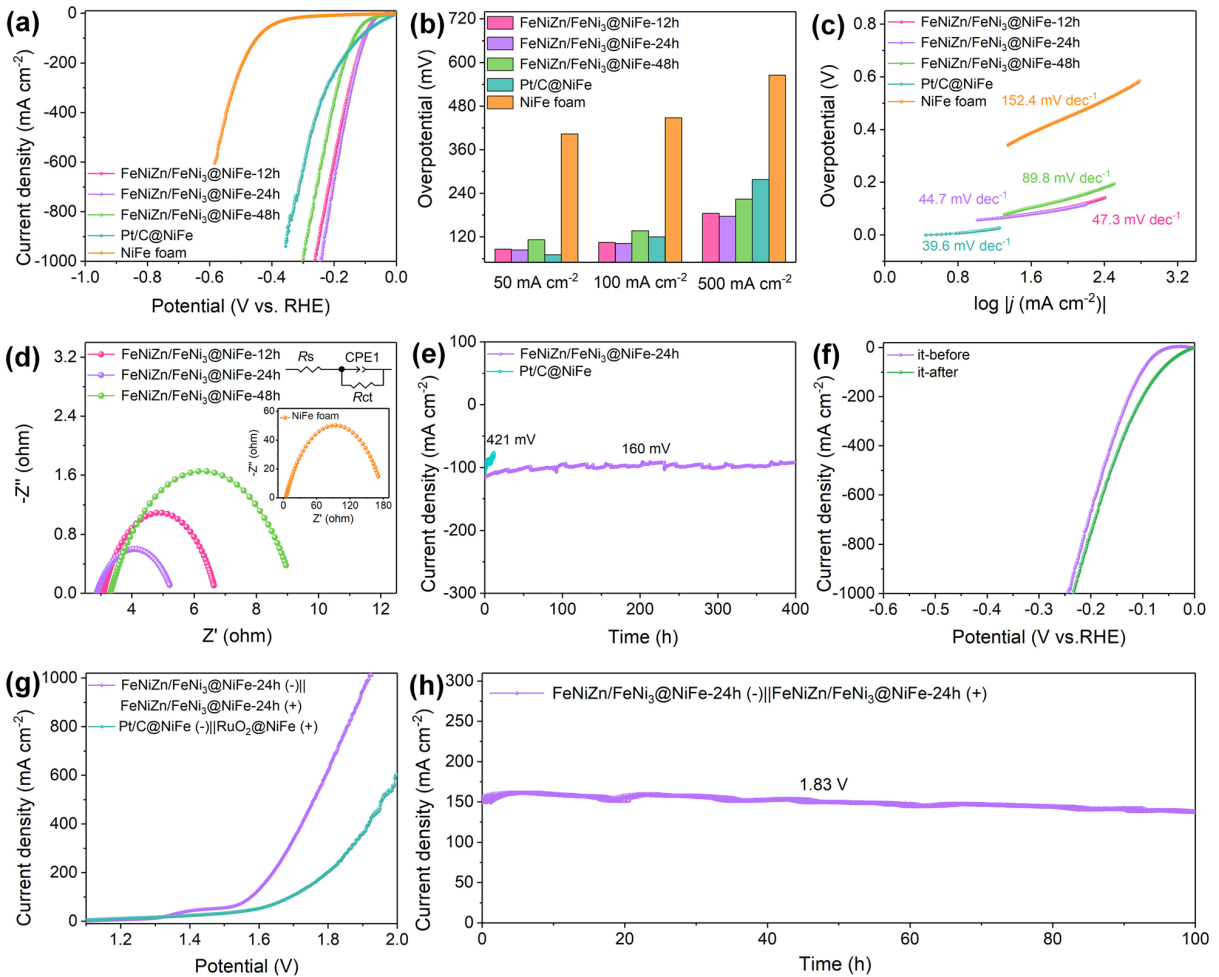
**a** LSV curves of FeNiZn/FeNi_3_@NiFe samples, Pt/C@NiFe, and NiFe foam in 1.0 M KOH solution at the scan rate of 5 mV s^−1^. **b** Overpotentials of all electrodes at different current densities. **c** Tafel plot. **d** EIS data of FeNiZn/FeNi_3_@NiFe samples and NiFe foam. **e** *i–t* curve under the overpotential of 160 and 421 mV for FeNiZn/FeNi_3_@NiFe-24 h and Pt/C@NiFe, respectively, and **f** LSV curves at a scan rate of 5 mV s^−1^ before and after *i–t* test for FeNiZn/FeNi_3_@NiFe-24 h toward HER. **g** LSV curves for overall water splitting with FeNiZn/FeNi_3_@NiFe-24 h and Pt/C@NiFe (−)||RuO_2_@NiFe (+) in a two-electrode device. **h** *i–t* test for FeNiZn/FeNi_3_@NiFe-24 h at a constant potential of 1.83 V in a two-electrode device

Aside from the excellent OER performances of FeNiZn/FeNi_3_@NiFe-24 h sample, it also shows the impressive HER performances. Figure 6a gives the LSV curves of FeNiZn/FeNi_3_@NiFe samples in 1.0 M KOH solution with those of Pt/C@NiFe and pure NiFe foam also tested for comparison. As shown in Fig. 6a, FeNiZn/FeNi_3_@NiFe-24 h sample displays the highest electrocatalytic activity toward HER with very small overpotential of 245 mV to reach the high current density of 1000 mA cm^−2^. Furthermore, it is clearly observed that the onset potential of Pt/C@NiFe toward HER is the lowest, but it became much larger than FeNiZn/FeNi_3_@NiFe-12 h and FeNiZn/FeNi_3_@NiFe-24 h samples when the current density is beyond to 70 mA cm^−2^. This is because the coated Pt/C particles on NiFe foam through the conductive binders are easily stripped down by the massive produced bubbles under the high current densities. Figure 6b provides the overpotential of all samples under the different current densities. FeNiZn/FeNi_3_@NiFe-24 h has the overpotential of 84 mV at 50 mA cm^−2^, which is 86, 112, and 403 mV less than FeNiZn/FeNi_3_@NiFe-12 h, FeNiZn/FeNi_3_@NiFe-48 h, and NiFe foam, respectively. At the current density of 100 and 500 mA cm^−2^, the overpotentials of FeNiZn/FeNi_3_@NiFe-24 h sample are only 102 and 177 mV, which are much lower than those of other electrodes. Moreover, the HER activity of FeNiZn/FeNi_3_@NiFe-24 h sample is superior to the comparable electrocatalysts reported previously as shown in Table S2, especially under the high current density [19, 20, 24, 26, 38, 44, 48–51, 53]. As stated above, it is regarded that the free-standing multimodal porous interpenetrating-phase heterostructure is responsible for the unique HER activity of FeNiZn/FeNi_3_@NiFe-24 h sample in virtue of high density of active sites and fluent mass transfer [3].

The inherent electrocatalytic properties of all samples toward HER are further unveiled by the Tafel plots as shown in Fig. 6c. The Tafel slope of FeNiZn/FeNi_3_@NiFe-24 h is 44.7 mV dec^−1^, which is slightly larger than the 39.6 mV dec^−1^ of Pt/C@NiFe catalyst, but smaller than those of other electrodes. The Tafel value of FeNiZn/FeNi_3_@NiFe-24 h means its Volmer-Heyrovsky mechanism during HER process with the adsorption/desorption of H intermediates as the rate-determination step [54, 55]. EIS data in Fig. 6d reveal that the *R*_ct_ values follows the same trend as the electrodes made by different dealloying time. Of note, FeNiZn/FeNi_3_@NiFe-24 h sample has the lowest *R*_ct_ value as small as 2.4 Ω, implying its more rapid electron transport and ion diffusion. In addition, FeNiZn/FeNi_3_@NiFe-24 h presents the excellent electrocatalytic durability toward HER under the overpotential of 160 mV with almost no decay in the long period of continuous 400 h, as illustrated in Fig. 6e. By contrast, the commercial Pt/C@NiFe required the overpotential as high as 421 mV to achieve the current density of 100 mA cm^−2^, while which underwent a significant activity decay with 79% of the initial current density retained upon merely testing for 12 h. This observation further proves that the particle-type catalyst is more likely to fall off at high current density than the self-supporting catalyst, thus resulting in poor catalytic stability [21]. More importantly, after the long-term test the LSV curve of FeNiZn/FeNi_3_@NiFe-24 h shifts to the forward, displaying the better HER activity with the decrease of the overpotential (Fig. 6f).

**Fig. 7:**
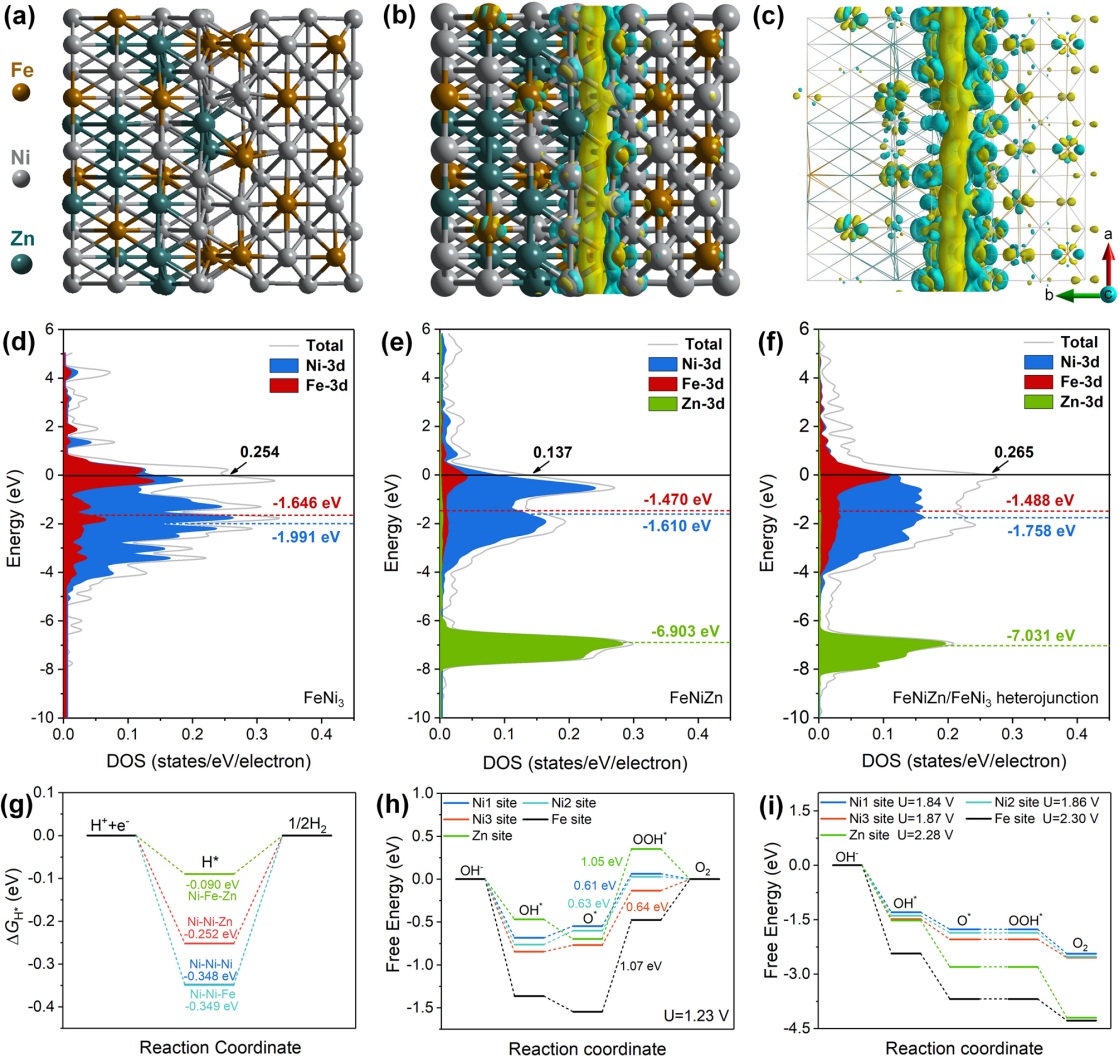
**a** The optimized structure of FeNiZn/FeNi_3_ heterojunction (orange balls are Fe, grey ones are Ni, and dark green ones are Zn). **b, c** Calculated charge density difference of FeNiZn/FeNi_3_ heterojunction. The calculated DOS of **d** FeNi_3_, **e** FeNiZn, and **f** FeNiZn/FeNi_3_ heterojunction, the dashed lines represent the d band center energy level. **g** The calculated Δ*G*_H*_ for the HER process at Ni–Ni–Ni, Ni–Ni–Zn, Ni–Ni–Fe, and Ni–Fe–Zn sites on FeNiZn/FeNi_3_@NiFe-24 h heterojunction. **h** Calculated free energy diagram for the OER at Fe, Ni, Zn sites at the applied potential of 1.23 V, and **i** the calculated free energy diagram for the OER on Fe, Ni, and Zn sites at different applied potentials

It is supposed that the high electrocatalytic stability of FeNiZn/FeNi_3_@NiFe-24 h sample originates from the robust self-supporting multilevel porous architecture. As shown in Fig. S8, it is clear that the FeNiZn/FeNi_3_@NiFe-24 h sample has almost no microstructure change as well as no phase structure transformation (Fig. S8a–c). It is worth mentioning that the pore channels became more penetrated through long period running in the alkaline solution accompanied with the decrease of Ni and Fe contents due to their slight dissolution as depicted in Fig. S8d. By comparison with the initial components, the reduced contents of Ni and Fe disclose that the FeNiZn alloy phase is more stable than FeNi_3_ intermetallic under the measurement conditions for HER. Meanwhile, the catalytic sites over FeNiZn (as shown in Fig. 7g) alloy increased on the surface, thus resulting in the activity improvement for HER upon long-term test. XPS data of Ni 2*p* and Zn 2*p* for the recycled sample is well agreement with those of pre-cycling sample (Fig. S9a, c). The little increase of Fe^3+^ suggests the slight oxidation of Fe^2+^ (Fig. S9b). It should be noted that the peak intensity of M–O bond evidently attenuated compared with those of M–OH and absorbed H_2_O, which implies that the major part of metal oxides on the surface has been reduced during hydrogen evolution process (Fig. S9d).

On the basis of the experimental observations above, the as-made FeNiZn/FeNi_3_@NiFe-24 h sample displays outstanding electrocatalytic performances toward both HER and OER in terms of low overpotential and long-term catalytic durability. It is interesting to utilize the FeNiZn/FeNi_3_@NiFe-24 h sample as both the OER and HER electrocatalysts to drive the water electrolysis. As shown in Fig. 6g, when the current density for hydrogen production reaches to 100 mA cm^−2^, the cell voltage only needs as small as 1.578 V, which is dramatically lower than the 1.691 V of Pt/C@NiFe (–)||RuO_2_@NiFe (+). Expressively, the voltage of merely 1.759 and 1.919 V is required to achieve the current density as high as 500 and 1000 mA cm^−2^ over FeNiZn/FeNi_3_@NiFe-24 h (+ //−), which is dramatically lower than that of Pt/C@NiFe (−)||RuO_2_@NiFe (+) (1.957 V@500 mA cm^−2^). We noted that the overall splitting performances of FeNiZn/FeNi_3_@NiFe-24 h electrode in alkaline electrolyzer is record-high compared with the similar and even the noble metal-containing materials in previous reports. For example, the IrNi-FeNi_3_/NF needs the 1.78 V to achieve the 500 mA cm^−2^; in contrast, our material only require 1.759 V [26]. As shown in Table S3, it is appealing that our catalyst outperforms other similar bifunctional ones for overall water splitting reported recently [20, 24, 26, 49, 51, 56–58]. Besides, under the cell voltage of 1.83 V for continuous running for 100 h in 1.0 M KOH, the hydrogen production current density over FeNiZn/FeNi_3_@NiFe-24 h (+ //−) retained the initial of 92.5%, exhibiting the steady and reliable hydrogen export (Fig. 6h).

### Density Functional Theory Calculations

DFT calculations were conducted for the sake of the origin of high bifunctional activity of FeNiZn and FeNi_3_ heterojunction toward HER and OER. The heterostructure model of FeNiZn and FeNi_3_ was constructed by using the (200) crystal planes with the different Ni coordinate sites involved, as displayed in Fig. 7a. Figure 7b, c gives the charge distribution in the FeNiZn/FeNi_3_ heterostructure, where the yellow region represents the electron accumulation, and the blue region represents the electron depletion calculated based on the equation: Δ*ρ* = *ρ*(FeNiZn/FeNi_3_)–*ρ*(FeNiZn)–*ρ*(FeNi_3_). It is clear that the FeNiZn section in the interface has the evident electron enrichment, revealing that the electrons in FeNi_3_ transfer to the neighboring FeNiZn. The calculated charge density difference in the FeNiZn/FeNi_3_ heterojunction in Table S4 further established this result, where the negative Bader charge for FeNi_3_ indicates its loss of electrons, further confirming the electron transfer from FeNi_3_ to FeNiZn. The density of states (DOS) of these models are also calculated as shown in Fig. 7d–f, from which it is evident that Ni, Fe, and Zn all have the metallic nature with zero band gaps, implying the fluent electron transport. Moreover, FeNiZn/FeNi_3_ has the largest DOS value at Fermi level, implying that the FeNiZn/FeNi_3_ heterostructure has much higher valence electron concentration. The *d* band center of Fe and Ni in FeNiZn/FeNi_3_ is synergistically modulated compared with those in FeNi_3_ and FeNiZn single counterpart. It is worthy to mention that too low or too high *d* band energy levels are disadvantageous toward the chemisorption process. The Sabatier principle uncovers that the moderate adsorption and desorption energies of reaction intermediates are crucial for the electrocatalytic efficiency [59–61]. Because higher *d* band energy levels decrease the electron filling of antibonding states, resulting in a too strong bond strength, which is unfavorable to the desorption of reaction intermediates. Likewise, the lower *d* band energy levels will greatly weaken the bond strength, possibly hindering the following catalytic process [62, 63]. The modulated *d*-band center of Ni, Fe, and Zn in FeNiZn/FeNi_3_ is more favorable for the moderate adsorption and desorption of reaction intermediates during water splitting as confirmed in the following data below.

Ni–Ni–Ni, Ni–Ni–Zn, Ni–Ni–Fe, and Ni–Fe–Zn sites on the FeNiZn/FeNi_3_ heterojunction were selected to explore the Δ*G*_H*_ to assess the HER activity of catalysts (Fig. S10). From Figs. 7g and S10, it is evident that Ni–Ni–Ni and adjacent Ni–Ni–Fe sites at the interface have the relatively large Δ*G*_H*_ values around − 0.348 and − 0.349 eV, indicating its stronger absorption and thus the high energy barriers for the subsequent formation of H_2_. For the Ni–Ni–Zn sites (Fig. S10b), during the calculation process the H will transfer onto the adjacent sites of Ni–Ni–Fe sites with the lower Δ*G*_H*_ around − 0.252 eV (Fig. S10c). This result revealed that the Ni–Ni–Fe site adjacent to Ni–Ni–Zn site has the higher HER activity compared with that adjacent to Ni–Ni–Ni site due to the substitution of one Zn atom to Ni. If one Zn atom further substitutes one Ni atom to form the Ni–Fe–Zn site, it is intriguing to find that the Δ*G*_H*_ can reach as small as − 0.090 eV. It can be concluded that the further introduction of Zn to near Ni–Fe sites can form much higher active sites, synergistically promoting the hydrogen evolution with more rapid reaction kinetics.

According to the OER mechanism, the absorption energy of OER intermediates (OH*, O*, and OOH*) were calculated over different Ni sites, Fe site, and Zn site on the interface of FeNiZn/FeNi_3_ heterojunction (Figs. S11–S12). From Figs. 7h and S13, it is evident that the conversion of O* to OOH* is the rate-determining step in the oxygen evolution for all sites on FeNiZn/FeNi_3_ heterojunction. Note that the O* to OOH* over three different Ni top sites on the interface has much low energy barriers around 0.61 to 0.64 eV at the applied potential of 1.23 V (Fig. 7h) and around 1.84 to 1.87 eV at the applied potential of 0 V (Fig. S13). However, it needs more than 1 eV over Fe and Zn top sites (Fig. 7h). From Fig. 7I, it can be found that the OER on Ni sites only need around the applied potentials of 1.84–1.87 V to achieve the spontaneous progress. In contrast, Fe site and Zn site for OER require much higher potentials of 2.30 and 2.28 V, respectively. This observation confirms that Ni sites on heterostructure surface possess more rapid reaction kinetics and thus much superior OER activity. In a word, FeNiZn/FeNi_3_ heterostructure owns high valence electron concentration and the optimized *d*-band center, as well as multiple highly active sites toward both HER and OER, thus resulting in outstanding bifunctional activity toward overall water splitting.

It is clearly found that FeNiZn/FeNi_3_@NiFe-24 h owns a series of exciting electrocatalytic performances toward water splitting as the bifunctional electrocatalyst. It is considered that its specific architecture and the extraordinary components contribute to the superior OER and HER activities. First, the bimodal nanoporous structure in situ built within the microporous NiFe foam increases the density of active sites and thus ensures highly efficient mass transfer, while the interconnected hollow channels can facilitate the electrolyte transportation as well as the full contact with active sites. Second, the integrated 3D multiscale porous architecture is conducive to boost the oxidation resistance and corrosion resistance, thus endowing the robust structure stability and catalytic durability. Third, the strong synergy between FeNiZn alloy and FeNi_3_ intermetallic generates the impelling electron modulation from the alloying effect, interface coupling, and structural benefits. These structural advantages can generate multiple synergistic actions to optimize the adsorption/desorption of reactants and thus the superior bifunctional activity toward HER and OER.

## Conclusions

In summary, one powerful dealloying strategy of Zn electrodeposition, annealing, and mild corrosion successfully in situ constructs bimodal porous interpenetrating FeNiZn and FeNi_3_ heterostructure anchored on the surface layer of 3D NiFe foam. On the account of self-supporting multiscale porous architecture, the intercrossing heterostructure, and strong synergy between FeNiZn alloy and FeNi_3_ intermetallic, the as-made FeNiZn/FeNi_3_@NiFe electrocatalyst displays superior electrocatalytic activities toward both HER and OER in terms of low overpotentials and the exceptional electrocatalytic durability, especially at large current density. In particular, the low cell voltage and steady hydrogen output under large current endow FeNiZn/FeNi_3_@NiFe prospective application in the industrial hydrogen production as the bifunctional electrocatalyst. The developed protocol may inspire great research interests to screen low cost, highly active, and highly stable intermetallic-based heterostructure electrocatalysts toward overall water splitting with the merits of scalable, handy, and massive preparation.

### Supplementary Information

Below is the link to the electronic supplementary material.

Supplementary file1 (PDF 3791 KB)
