# Supplementary material for: Duplex Interpenetrating-Phase FeNiZn and FeNi3 Heterostructure with Low-Gibbs Free Energy Interface Coupling for Highly Efficient Overall Water Splitting
Source: Nanomicro Lett. 2023 Apr 10;15:95. doi: 10.1007/s40820-023-01066-w (PMC10086094; doi:10.1007/s40820-023-01066-w)
Supplement: Supplementary file 1 — Supplementary file1 (PDF 3791 KB) [file 40820_2023_1066_MOESM1_ESM.pdf]

Supporting Information for

Duplex Interpenetrating-Phase FeNiZn and $FeNi_3$ Heterostructure with Low-Gibbs Free Energy Interface Coupling for Highly Efficient Overall Water Splitting

Qiuxia Zhou[1, 2], Caixia Xu[1, *], Jiagang Hou[3], Wenqing Ma[1], Tianzhen Jian[1], Shishen Yan[1], and Hong Liu[1, 4, *]

[1]Institute for Advanced Interdisciplinary Research (iAIR), Spintronics Institute, Collaborative Innovation Center of Technology and Equipment for Biological Diagnosis and Therapy in Universities of Shandong, School of Chemistry and Chemical Engineering, University of Jinan, Jinan 250022, People's Republic of China

[2]School of Medical Information and Engineering, Southwest Medical University, Luzhou 646000, People's Republic of China

[3]Kyiv College at Qilu University of Technology, Qilu University of Technology, Shandong Academy of Sciences, Jinan 250353, People's Republic of China

[4]State Key Laboratory of Crystal Materials, Shandong University, Jinan 250100, People's Republic of China

*Corresponding authors. E-mail: chm_xucx@ujn.edu.cn (Caixia Xu); ifc_liuh@ujn.edu.cn (Hong Liu)

Supplementary Figures and Tables

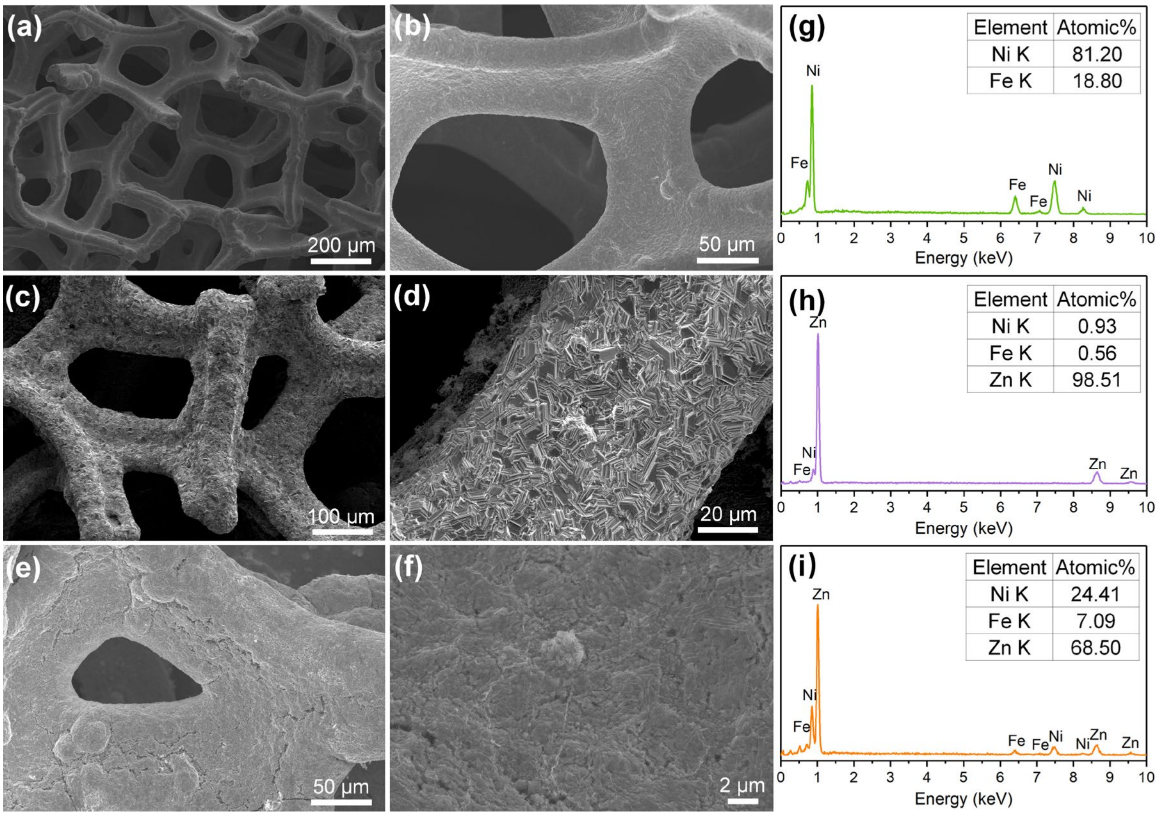

Fig. S1 SEM images of **a, b** NiFe foam, **c, d** Zn@NiFe, **e, f** the annealed Zn@NiFe sample, and the EDS data of **g** NiFe foam, **h** Zn@NiFe, and **i** the annealed Zn@NiFe sample

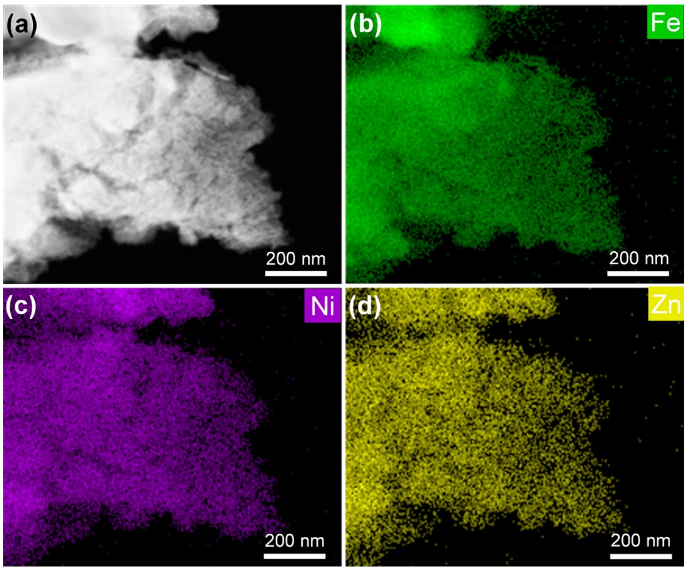

Fig. S2 TEM image **a** and the corresponding the EDS mapping images of **b** Fe, **c** Ni, and **d** Zn for FeNiZn/$FeNi_3$@NiFe-24 h sample

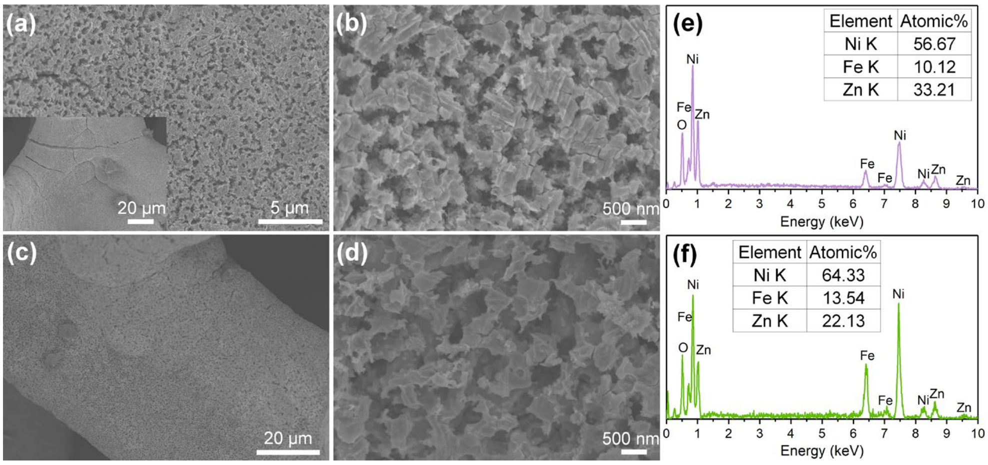

Fig. S3 SEM images of **a, b** FeNiZn/$FeNi_3$@NiFe-12 h and **c, d** FeNiZn/$FeNi_3$@NiFe-48 h, the EDS data of **e** FeNiZn/$FeNi_3$@NiFe-12 h and **f** FeNiZn/$FeNi_3$@NiFe-48 h samples

As displayed in Fig. S3, we further explored the effect of the different corrosion time on the resulting structure. At the corrosion time of 12 h, it can be found that there are some ligaments with the size of 500 nm intercrossed to form the network framework as well a number of uneven pits with the size of several hundred nanometers distributed on the sample surface own to the dissolution of Zn atoms (Fig. S3a, b). Upon extending the corrosion time to 24 h, it is clearly seen that a uniform porous structure was observed on the surface of the sample and the the ligament size was reduced to 300 nm due to the increasing dissolution of Zn atoms (Fig. 3a, b). Further extending the corrosion time to 48 h, resulted sample also shows a similar porous structure (Fig. S3c, d). EDS data (Fig. S3e, f and Fig. 3c) suggest that the residual contents of Zn gradually decreases as the corrosion time increases from 12 to 24 h. However, the residual Zn content little significant change from 24 to 48 h because the more reactive Zn atoms from the Ni_2Zn_{11} and $Fe_{6.8}Zn_{3.2}$ phases have been almost completely etched. This result proves that the pure Zn, Ni_2Zn_{11}, and $Fe_{6.8}Zn_{3.2}$ phases disappeared at the corrosion time of 24 h, which is consistent with the XRD results in Fig. 2c, d. The larger pores were generated on account of the etching of the pure zinc phase, which the smaller pores formed due to the corrosion of Zn from Ni_2Zn_{11} and $Fe_{6.8}Zn_{3.2}$ alloys. Based on the results above, it is shown that upon etching Zn from the different phase of precursor alloy, the interpenetrating-phase

FeNiZn alloy and $FeNi_3$ intermetallic heterostructure was generated with bimodal porous structure. Besides, the phase structure and residual content of Zn remained almost unchanged in the 48 h, which proves that FeNiZn alloy and $FeNi_3$ intermetallic have high structural stability and good corrosion resistance in alkaline solution, which provides the substantial basis for their application in electrocatalytic water splitting.

Table S1 The OER performances comparison of FeNiZn/$FeNi_3$@NiFe-24 h sample with other similar electrocatalysts in 1.0 M KOH solution

Material	Tafel slope ($mV dec^{-1}$)	η (mV) @j ($mA cm^{-2}$)	Substrate	Refs.
NiFe/NF	76.2	230@10<br>289@50	NF	[38]
$FeNi_3$@GCDs-10	48.7	238@10	GCE	[47]
$Ni_{30}Fe$ alloy/Ni foil	41	390@300	Ni foil	[48]
redox-NiFe foam	38	294@100	$FeNi_3$ foam	[39]
Ni_3FeN/Ni_3Fe	51	250@10	GCE	[19]
$MoNiFeS_x$@$FeNi_3$	72.8	142@10	$FeNi_3$ foam	[49]
NiFe nanotube arrays	45	236@10<br>371@400	Au/Ni film	[50]
Ni-FeO_x/$FeNi_3$	103.0	269@50<br>405@1000	NF	[24]
$FeNi_3$@FeNi LDH	51.4	225@50	NF	[51]
$FeNi_3$-$FeNi_3N$	53	260@10	GCE	[20]
IrNi-$FeNi_3$/NF	36.01	270@100<br>300@500<br>330@1000	NF	[26]
FeNiZn/$FeNi_3$@NiFe-24 h	45.7	244@50<br>258@100<br>315@500<br>367@1000	NiFe foam	This work

GCDs: graphene carbon dots; GCE: glass carbon electrode; NF: nickel foam; LDH: layered double hydroxide

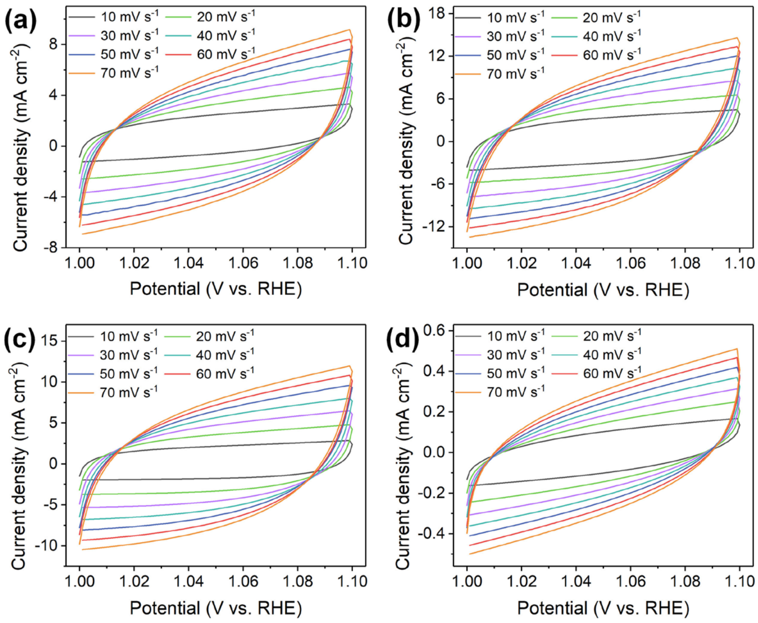

Fig. S4 CV curves of **a** FeNiZn/$FeNi_3$@NiFe-12 h, **b** FeNiZn/$FeNi_3$@NiFe-24 h, **c** FeNiZn/$FeNi_3$@NiFe-48 h, and **d** NiFe foam at different scan rates of 10, 20, 30, 40, 50, 60, and 70 mV s^{-1}

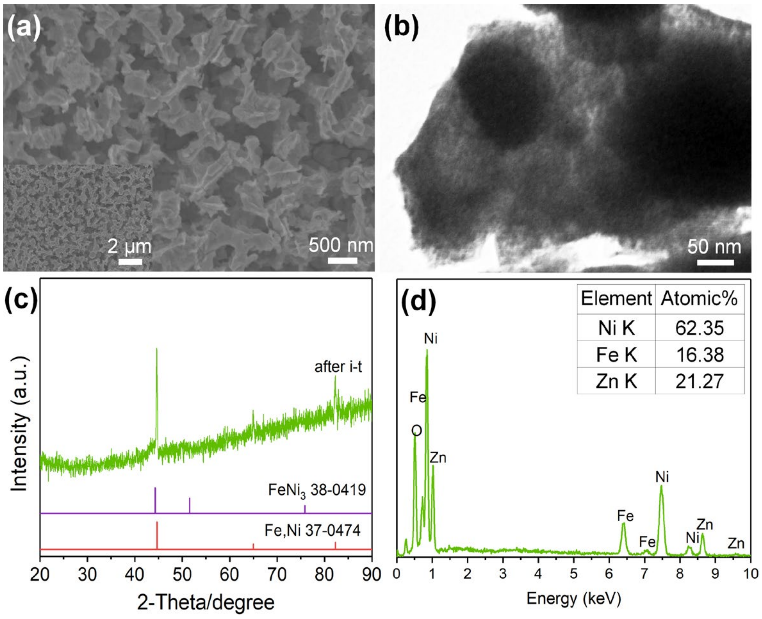

Fig. S5 a SEM image, **b** TEM image, **c** XRD pattern, and **d** EDS data of FeNiZn/$FeNi_3$@NiFe-24 h sample after long term OER test in 1.0 M KOH solution for 400 h

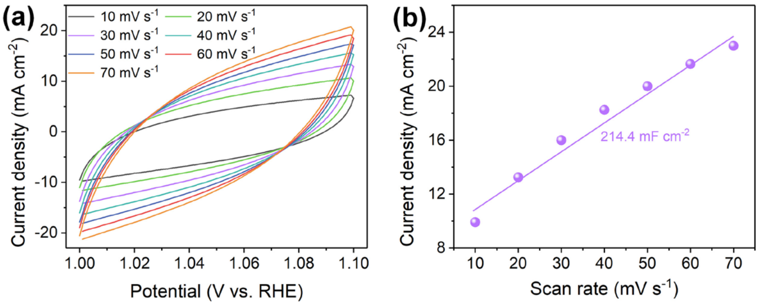

Fig. S6 a CV curves at different can rates of 10, 20, 30, 40, 50, 60, and 70 mV s^{-1}, **b** the capacitive current at 1.05 V vs. RHE with different scan rates for FeNiZn/$FeNi_3$@NiFe-24 h

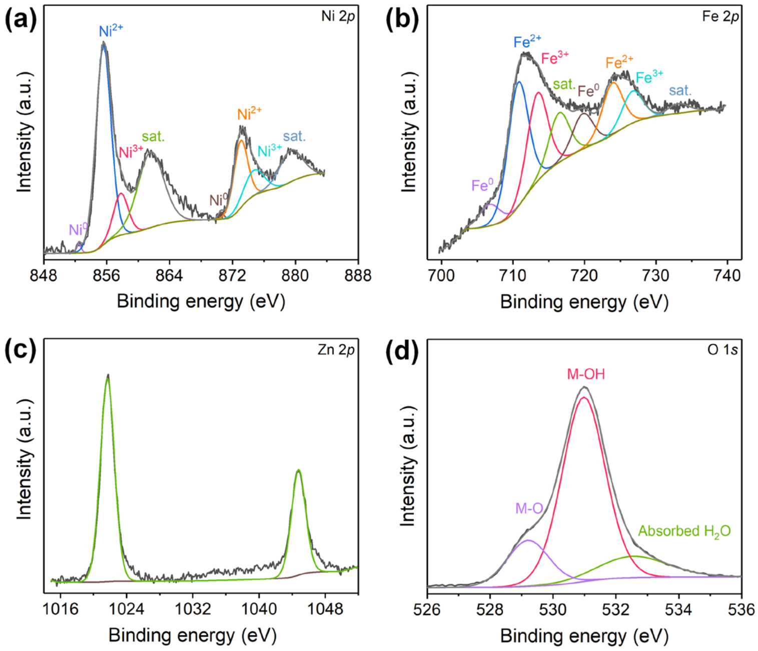

Fig. S7 XPS data of a Ni 2*p*, b Fe 2*p*, c Zn 2*p*, and d O 1*s* for FeNiZn/$FeNi_3$@NiFe-24 h sample after long term OER test in 1.0 M KOH solution for 400 h

Table S2 The HER performances comparison of FeNiZn/$FeNi_3$@NiFe-24 h sample with some similar electrocatalysts reported previously in 1.0 M KOH solution

Material	Tafel slope (mV dec^{-1})	η (mV) @j (mA cm^{-2})	Substrate	Refs.
NiFe/NF	158.9	59@10<br>175@50	NF	[38]
NiCo alloy foam	49	100@40	Ni foil	[48]
Ni_3FeN/Ni_3Fe	98.1	125@10	GCE	[19]
nanoporous NiMnFeMo	49	67@100<br>178@500<br>290@1000	nanoporous NiMnFeMo	[44]
amorphous NiFe NAs-NF	147	181@10	NF	[53]
$MoNiFeS_x$@$FeNi_3$	57.5	67@10	$FeNi_3$ foam	[49]
NiFe alloy NAs	78	164@10<br>360@400	Au/Ni foil	[50]
Ni-FeO_x/$FeNi_3$	44.6	71@50<br>272@1000	NF	[24]
$FeNi_3$@FeNi LDH	122	106@10	NF	[51]
$FeNi_3$-$FeNi_3N$	55.9	51@10<br>86@20<br>215@100	GCE	[20]
IrNi-$FeNi_3$/NF	66.95	138.9@100<br>248.6@500<br>288@1000	NF	[26]
FeNiZn/$FeNi_3$@NiFe-24 h	44.7	84@50<br>102@100<br>177@500<br>245@1000	NiFe foam	This work

NAs: Nanotubes

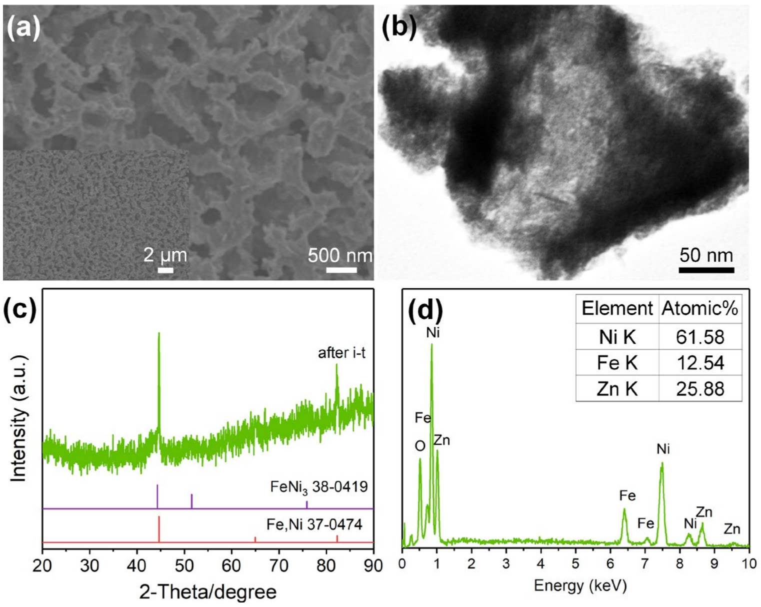

Fig. S8 **a** SEM image, **b** TEM image, **c** XRD pattern, and **d** EDS data of FeNiZn/$FeNi_3$@NiFe-24 h sample after long term HER test in 1.0 M KOH solution for 400 h

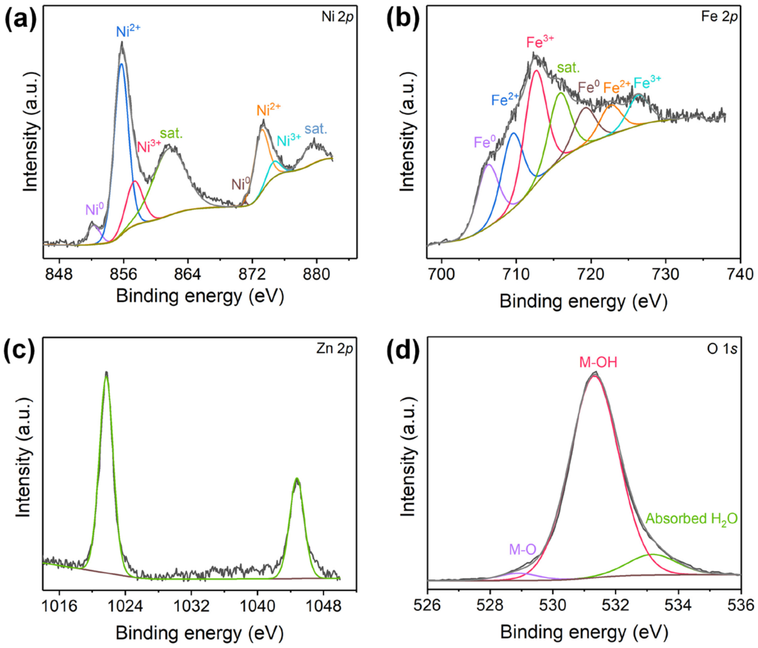

Fig. S9 XPS data for **a** Ni 2*p*, **b** Fe 2*p*, **c** Zn 2*p*, and **d** O 1*s* of FeNiZn/$FeNi_3$@NiFe-24 h sample after long term HER test in 1.0 M KOH solution for 400 h

Table S3 The performances comparison of FeNiZn/$FeNi_3$@NiFe-24 h constructed electrolyzer with some representative bifunctional catalysts in basic solutions

Material	η (V) @j (mA cm^{-2})	Substrate	Refs.
Ni-FeO_x/$FeNi_3$	1.58@50<br>1.80@500	NF	[24]
MoNiFeS_x@$FeNi_3$	1.50@10	$FeNi_3$ foam	[49]
NiFeCoS_x@$FeNi_3$	1.54@10	$FeNi_3$ foam	[56]
Fe-Ni_3S_2@$FeNi_3$-8	1.50@10	$FeNi_3$ foam	[57]
$FeNi_3$/NiFeO_x	1.55@10<br>2.0@100	NF	[58]
$FeNi_3$@FeNi LDH	1.68@10	NF	[51]
$FeNi_3$-$FeNi_3N$	1.5@10	GCE	[20]
IrNi-$FeNi_3$/NF	1.47@10<br>1.78@500	NF	[26]
FeNiZn/$FeNi_3$@NiFe-24 h	1.578@100<br>1.759@500<br>1.919@1000	NiFe foam	This work

Table S4 The calculated bader charge of FeNiZn/$FeNi_3$ heterojunction

	Q(FeNiZn)	Q($FeNi_3$)	ΔQ
FeNiZn/$FeNi_3$	0.091	-0.091	0.091

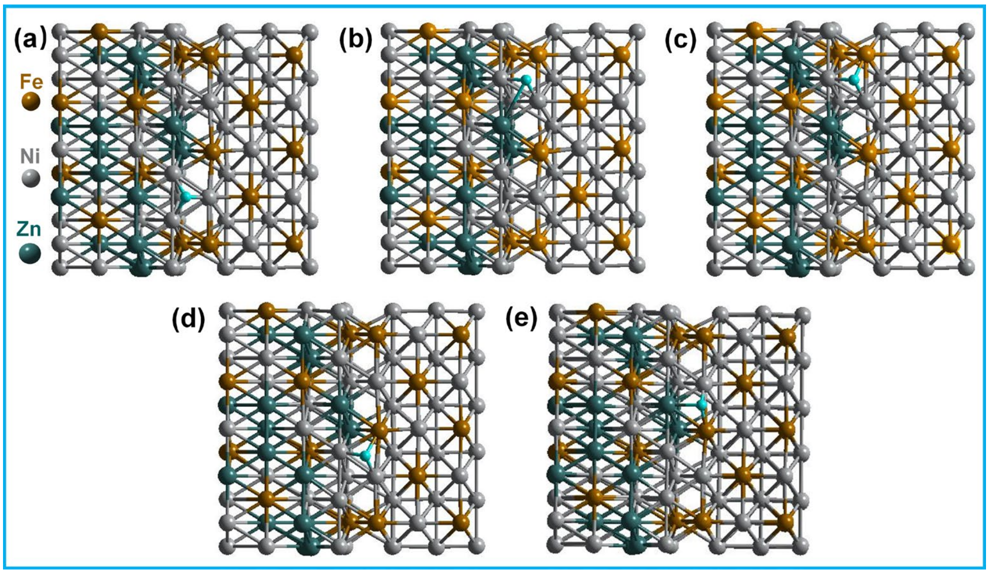

Fig. S10 The optimized structure of hydrogen adsorbed on **a** Ni-Ni-Ni site, **b** the theoretical constructed structure of hydrogen adsorbed on Ni-Ni-Zn site and **c** the final stable site for Ni-Ni-Fe site, **d** Ni-Ni-Fe site, and **e** Ni-Fe-Zn site. Orange balls are Fe, grey ones are Ni, dark green ones are Zn, and the cyan is H

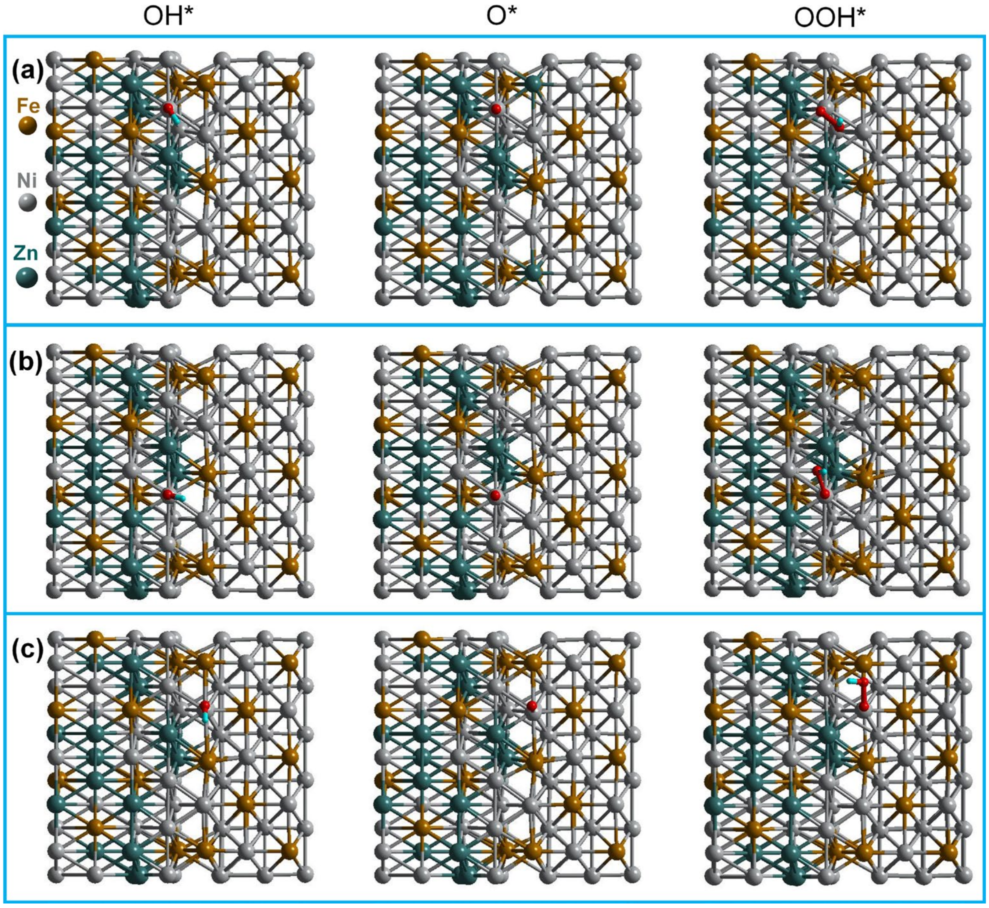

Fig. S11 The optimized structural configurations of FeNiZn/$FeNi_3$ heterojunction for the feasible adsorption of OER intermediates (OH*, O*, and OOH*) at **a** Ni1, **b** Ni2, and **c** Ni3 site. Orange balls are Fe, grey ones are Ni, dark green ones are Zn, the cyan is H, and the red is O

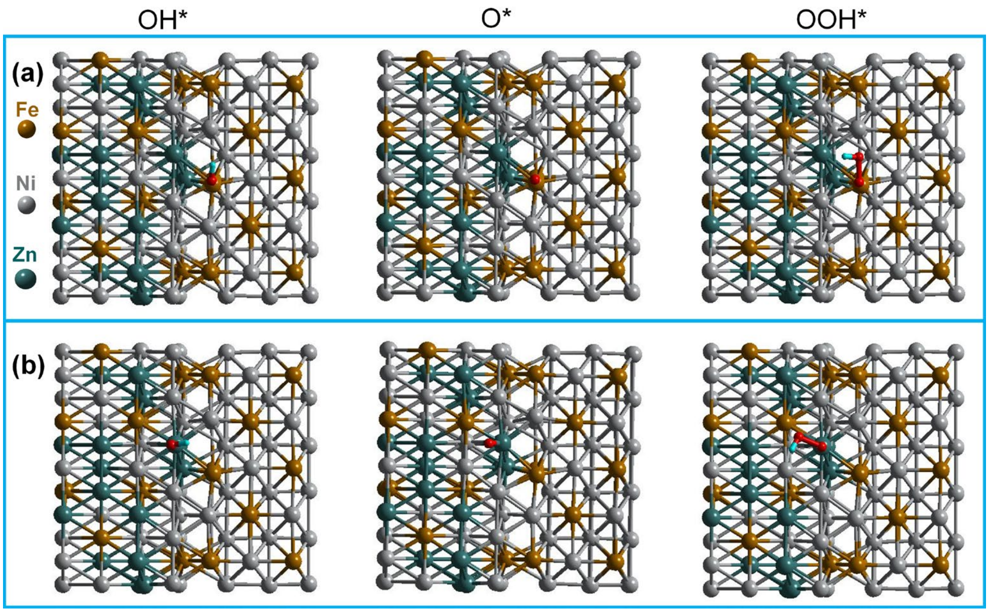

Fig. S12 The optimized structural configurations of FeNiZn/$FeNi_3$ heterojunction for the feasible adsorption of OER intermediates (OH*, O*, and OOH*) at **a** Fe and **b** Zn site. Orange balls are Fe, grey ones are Ni, dark green ones are Zn, the cyan is H, and the red is O

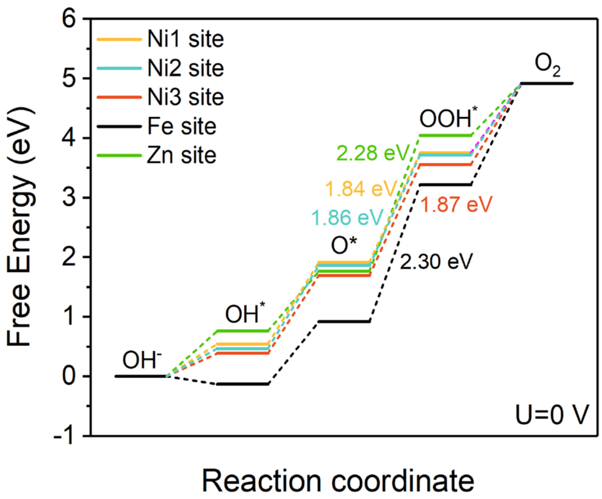

Fig. S13 The calculated free energy diagram for the OER at Fe, Ni, and Zn site at the applied potential of 0 V on FeNiZn/$FeNi_3$ heterojunction
